# S‐9‐PAHSA Protects Against High‐Fat Diet‐Induced Diabetes‐Associated Cognitive Impairment via Gut Microbiota Regulation

**DOI:** 10.1111/cns.70417

**Published:** 2025-05-05

**Authors:** Shanshan Huang, Xinru Wang, Meng Wang, Jinhong Lin, Jiaoqi Ren, Chenyu Lu, Jiayu Fu, Yanli Zhang, Xuechun Wang, Jichang Xiao, Jingchun Guo, Houguang Zhou

**Affiliations:** ^1^ Department of Geriatric of Huashan Hospital, National Clinical Research Center for Aging and Medicine Fudan University Shanghai China; ^2^ State Key Laboratory of Fluorine and Nitrogen Chemistry and Advanced Materials Chinese Academy of Sciences Shanghai China; ^3^ Department of Translational Neuroscience, Jing'an District Centre Hospital of Shanghai, State Key Laboratory of Medical Neurobiology and MOE Frontiers Center for Brain Science, and Institutes of Brain Science Fudan University Shanghai China

**Keywords:** diabetes‐associated cognitive impairment, gut microbiota, PI3K/AKT/mTOR, S‐9‐PAHSA, synaptic dysfunction

## Abstract

**Aim:**

Diabetes‐associated cognitive impairment (DACI) is a common complication of Type 2 diabetes mellitus (T2DM), with its mechanisms and treatments for DACI remaining incompletely clarified. This study investigated the protective efficacy of the novel lipid S‐enantiomer of 9‐palmitic acid esters of hydroxy stearic acids (S‐9‐PAHSA, S9P) in a high‐fat diet‐induced DACI mouse model.

**Methods:**

Mice were randomly assigned to three groups: normal diet (ND), high‐fat diet (HFD), and HFD + 30 mg/kg/day S9P (HFD + S9P). Fasting blood glucose (FBG), intraperitoneal glucose tolerance test (IPGTT), and insulin tolerance test (ITT) were conducted to assess blood glucose homeostasis. Morris Water Maze and Y maze tests evaluated cognitive function, and neuronal status was examined through pathological analysis, Golgi staining, and transmission electron microscopy (TEM). Colonic barrier integrity was assessed using periodic acid–Schiff and Alcian blue staining (AB‐PAS) and immunohistochemistry (IHC) staining. Intestinal microbiota composition was analyzed by 16S rDNA sequencing, and serum metabolic characteristics were determined by metabolomics sequencing.

**Results:**

S9P improved glucose homeostasis and alleviated cognitive decline in DACI mice. It also mitigated neuronal damage, dendritic degeneration, and synaptic damage, while restoring colonic barrier integrity and ameliorating gut microbiome imbalances, insulin resistance, and lipid imbalance. Additionally, S9P regulated metabolite profiles and the PI3K/AKT/mTOR signaling pathways, and reduced astrocyte activation and neuroinflammatory responses in the hippocampus of HFD‐induced DACI mice.

**Conclusion:**

S9P had a protective effect against HFD‐induced diabetic cognitive impairment closely related to the modulation of the gut–brain axis, suggesting that S9P has the potential to become a new therapeutic approach for DACI.

## Graphical abstract

This study reveals S9P's protective effect on HFD‐induced cognitive decline. By bolstering intestinal barriers, refining gut microbiome balance, and stabilizing serum metabolism, S9P mitigates hippocampal inflammation and synaptic damage. S9P emerges as a promising therapeutic agent for cognitive deficits linked to high‐fat diets, targeting the gut–brain axis.

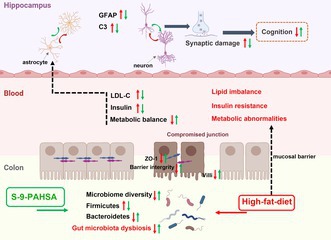

## 1 Introduction

Type 2 diabetes mellitus (T2DM) is a chronic metabolic disorder marked by dysregulated glucose and lipid metabolism and persistent systemic inflammation [1]. These metabolic disturbances and chronic inflammation not only inflict widespread damage on peripheral organs but also exert detrimental effects on the central nervous system (CNS) [2, 3]. Growing evidence indicates a strong association between T2DM and an elevated risk of cognitive impairment [4, 5]. Patients with T2DM frequently exhibit significant deficits in memory, executive function, and attention, collectively referred to as diabetes‐associated cognitive impairment (DACI) [6]. However, the pathophysiological mechanisms underlying DACI remain incompletely understood and warrant further investigation.

The pathological mechanism of DACI is complex, involving multiple factors such as hyperglycemia‐induced toxicity [7, 8], insulin resistance [9], oxidative stress [10], and dysregulation of the gut–brain axis [11]. Insulin not only regulates peripheral glucose metabolism but also plays a crucial signaling role in the central nervous system, where it modulates neuronal energy metabolism and synaptic plasticity [12]. A 5‐year longitudinal MRI study indicated that T2DM may accelerate impairments in neurovascular coupling in certain brain regions, leading to memory decline [13]. Recent studies indicate that the gut–brain axis plays a significant role in DACI [11]. Evidence from animal studies has shown that mice on long‐term HFD frequently develop imbalances in gut microbiota, particularly changes in the Bacteroidetes‐to‐Firmicutes ratio, which disrupt the microecological balance typical of healthy states [14]. The elevated proportion of Firmicutes relative to Bacteroidetes triggers systemic low‐grade inflammation, exacerbates insulin resistance, and contributes to metabolic dysregulation [15]. This imbalance disrupts the gut–metabolism–brain axis, causing negative effects on cognitive function [14]. The inflammation and metabolic disturbances associated with this dysbiosis may increase blood–brain barrier permeability and promote neuroinflammation, compounding the impact on cognitive decline.

PAHSAs (Palmitic Acid Esters of Hydroxy Stearic Acids) are novel endogenous lipids within the palmitate subfamily of hydroxy stearate esters, naturally present in mammals and predominantly located in adipose tissue [16, 17, 18, 19]. Composed of palmitic acid and hydroxystearic acid linked by an ester bond, PAHSA was found at lower levels in the circulation and adipose tissue of individuals with insulin resistance, showing a strong positive correlation with insulin sensitivity [16, 20, 21]. Similarly, insulin‐resistant mice exhibit decreased PAHSAs levels in subcutaneous fat and serum [22]. Dietary or pharmacological interventions to increase PAHSAs levels may therefore offer a promising approach to alleviate insulin resistance. Previous studies have shown that exogenous PAHSAs supplementation significantly reduces insulin resistance and enhances glucose metabolism in vivo [23, 24]. Additionally, A study has shown that PAHSAs can influence the gut microbiota, which is beneficial for their metabolic effects in HFD‐fed mice [25]. PAHSAs can be classified into isomers, such as 9‐PAHSA and 10‐PAHSA, based on the position of the hydroxyl group in hydroxystearic acid [18]. These stereoisomers can exist in R or S configurations depending on the branching carbon, and different configurations may influence their interactions with receptors or enzymes, leading to distinct biological effects [26]. Research indicates that 9‐PAHSA improves insulin sensitivity and glucose tolerance in mice by enhancing glucose‐stimulated insulin secretion (GSIS), insulin‐stimulated glucose uptake, and insulin action, thereby reducing hepatic glucose production and adipose tissue inflammation [27]. Two enantiomers of 9‐PAHSA, R‐9‐PAHSA and S‐9‐PAHSA (S9P) have been identified in mouse white adipose tissue. Notably, S9P shows a significant advantage over R‐9‐PAHSA in promoting GSIS and insulin‐stimulated glucose uptake, indicating its greater potential for improving insulin sensitivity and glucose metabolism [28].

Therefore, we synthesized S9P to investigate its therapeutic potential and mechanisms in the DACI model. We examined the cortical and hippocampal pathology, gut microbiota changes, serum metabolites, and lipid metabolism to investigate S9P's effects on diabetic cognitive impairment through the gut–brain axis, providing insights for novel therapeutic approaches.

## 2 Materials and Methods

### 2.1 Preparation of S9P

9‐bromo‐1‐nonene underwent a Grignard reaction, followed by reactions with decanal, palmitic anhydride, and ozonolysis to yield 9‐PAHSA, which was purified and resolved to obtain the pure S‐enantiomer with a purity of 99% (Figure S1A). The structure of the product was confirmed by hydrogen nuclear magnetic resonance (^1^H NMR) spectroscopy, and the corresponding NMR spectrum is shown in Figure S1B.

### 2.2 Animals and Experimental Design

**FIGURE 1:**
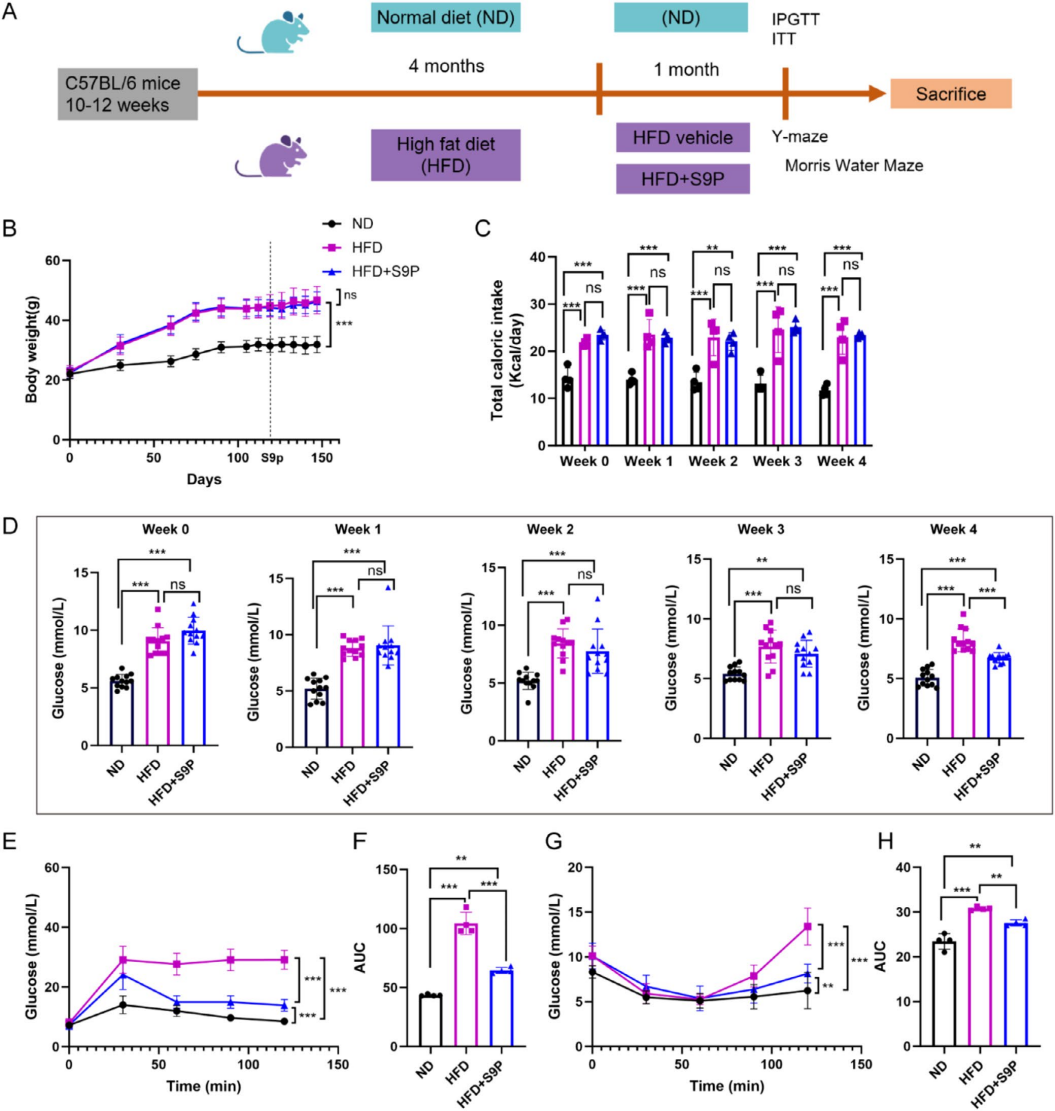
Effects of S9P for a month on body weight and glucose metabolism in HFD‐induced DACI mice. (A) Mice grouping and S9P administration. (B) Body weight trend and change of mice in different groups, *n* = 20. (C) Average caloric intake per cage since S9P supplementation weekly (4 cages per group, with five mice per cage). (D) Weekly fasting blood glucose levels of mice in each group following S9P administration, *n* = 12. (E, F) IPGTT and AUC of IPGTT, *n* = 12. (G, H) ITT and AUC of ITT, *n* = 12. Data were displayed as the mean ± SEM. *p < 0.05, **p < 0.01, and ***p < 0.001.

Healthy male C57BL/6 mice (10–12 weeks) were obtained from the Shanghai Rodent Laboratory Animal Center. The mice were housed under standard conditions (22°C, 12‐h light–dark cycle) with free access to food and water. They were randomly assigned into three experimental groups (*n* = 20 per group): normal diet (ND), high‐fat diet (HFD), and S9P intervention (HFD + S9P). A model of DACI was induced by feeding the mice a high‐fat diet (60% fat, 20% carbohydrate, and 20% protein) for 5 months. Mice in the HFD + S9P group received a daily dose of S9P solution (30 mg/kg/day) [24] after 4 months of the HFD, with fasting blood glucose (FBG) levels monitored weekly. After 5 months, all the mice were subjected to glucose tolerance tests and behavioral experiments; then, the mice were sacrificed under anesthesia with 3% sodium pentobarbital (45 mg/kg, ip), and serum samples, brain tissue, colon tissue, and fecal matter were collected for subsequent analysis. Figure 1A shows the procedures for grouping and S9P administration.

### 2.3 Fasting Blood Glucose (FBG) Measurement, Intraperitoneal Glucose Tolerance Test (IPGTT), and Insulin Tolerance Test (ITT)

Mice blood glucose levels were assessed using a Roche glucometer with C‐type strips (Roche Diagnostics Switzerland), with fasting blood glucose (FBG) measured after 4 months on a high‐fat diet and weekly thereafter, following a 14‐h fast with water access. FBG values were determined from tail vein samples. Both the intraperitoneal glucose tolerance test (IPGTT) and the insulin tolerance test (ITT) were conducted after the S9P intervention. These tests were performed once, 30 days postintervention, and were not carried out before the intervention. For IPGTT, mice fasted for 14 h and received a 40% glucose injection at 2 g/kg, with glucose levels measured at 0, 30, 60, and 120 min postinjection for IPGTT calculation. In the ITT, mice fasted overnight and were injected with insulin at 0.75 IU/kg, with plasma glucose measured at the same time points as IPGTT.

### 2.4 Morris Water Maze (MWM) Test

The MWM test assessed spatial learning and memory in mice by placing them in a circular pool with opaque, temperature‐controlled water. Mice were trained for 5 days to locate a submerged platform using spatial cues, with recorded latency to reach the platform. On the test day, the platform was removed, and the number of crossings over the platform location and time spent in the target quadrant was tracked using EthoVision XT 8.5 (Noldus, Netherlands).

### 2.5 Y Maze Test

The Y‐maze test was utilized to assess short‐term memory, spontaneous alternation behavior, and working memory in experimental animals. Mice were placed at the junction and allowed to explore for 8 min, with spontaneous alternation measured by consecutive entries into three arms. Working memory was calculated as the ratio of actual to maximum possible alternations. Movement and data were analyzed using EthoVision XT14 (Noldus, Netherlands).

### 2.6 Hematoxylin–Eosin (H&E) Staining and Nissl Staining

We evaluated hippocampal and cortical neuronal structure and integrity in mice through Hematoxylin and Eosin (H&E) and Nissl staining. For Nissl staining, sections were dehydrated in xylene and ethanol, stained with 1% tar violet or thionine, differentiated in 70% alcohol, and mounted in DPX after final dehydration and clearing. H&E staining involved staining with Harris hematoxylin, differentiation in hydrochloric acid alcohol, bluing in ammonia water, and eosin counterstaining, followed by dehydration, xylene clearing, and neutral gum mounting. Imaging was performed under a BX53 light microscope (Olympus, Japan).

### 2.7 Golgi Staining and Neuronal Morphological Analysis

The brains were stained using the FD Rapid Golgi Staining Kit (PK401, FD Neurotech, USA) following the manufacturer's protocol. Coronal brain sections (100 μm thick) were cut using a vibratome and mounted on gelatin‐coated slides. The sections were dried, dehydrated through a graded ethanol series, and cleared in xylene. Morphological analysis was performed after imaging with a BX53 light microscope (Olympus, Japan). Granule neurons in the CA1 region of the hippocampus and cortex were reconstructed and analyzed using ImageJ (National Institutes of Health, USA) and Sholl analysis.

### 2.8 Immunofluorescence (IF) Staining

For immunofluorescence staining, paraffin‐embedded brain sections were blocked with 3% BSA for 1 h and subsequently incubated overnight at 4°C with primary antibodies against Occludin, GFAP, C3, and SYN (Table S1). After washing with PBS, the sections were incubated with the corresponding secondary antibodies for 1 h at room temperature. Nuclei were counterstained with 4',6‐diamidino‐2‐phenylindole (DAPI). The stained sections were then examined using a microscope (Nikon, Japan). 3D reconstruction of astrocytes, synapses, and C3 was completed by Imaris software (Bitplane, Switzerland).

### 2.9 Alcian Blue‐Periodic Acid Schiff (AB‐PAS) Staining and Immunohistochemistry (IHC) Staining

The colonic segments were immediately fixed in 4% paraformaldehyde in 0.1 M phosphate‐buffered saline (pH 7.4, 4°C) for 48 h and embedded in paraffin for sectioning (5 μm cross section). The tissue sections were stained with periodic acid‐Schiff (AB‐PAS). In the colon, at least 30 random fields in six sections of each sample with AB‐PAS staining were photographed at ×400 magnification with a microscope (Olympus BX51, Japan).

### 2.10 Transmission Electron Microscopy (TEM)

After transcardial perfusion with saline, 1 mm^3^ hippocampus tissue and 2 mm colonic segments were cut and fixed in a solution of 2% glutaraldehyde and 2% formaldehyde in 0.1 M phosphate buffer (pH 7.4). Subsequent steps were performed as before [29]. Ultrastructural observations were conducted using a Hitachi‐H7650 transmission electron microscope (Hitachi, Japan).

### 2.11 Gut Microbiota Analysis

Cecal contents from mice were collected and stored at −80°C before being sent to Lianchuan Biotechnology (Hangzhou, China) Co. Ltd. for microbiome DNA extraction and 16S rDNA sequencing. Raw sequencing data underwent quality filtering, and high‐quality data were processed using OmicStudio for further analysis. A comprehensive analysis was then conducted to assess the intestinal microbiota's abundance, diversity, similarity, and compositional profiles.

### 2.12 Metabolomics Profiling

Untargeted serum metabolomics was conducted by the Vanquish Flex UHPLC system (Thermo, USA) 200 μL serum was vortexed with 400 μL methanol, followed by supernatant collection. Samples were vacuum‐concentrated to dryness, then redissolved in an 80% methanol solution containing 2‐chloroaniline, and aliquoted into vials for LC–MS analysis. The chromatography was executed with a gradient elution protocol postsystem equilibration. Raw data underwent preprocessing via the XCMS, CAMERA, and metaX toolboxes in R studio, with metabolite identifications referencing the KEGG and HMDB databases. Only metabolite signatures consistently detected in at least 50% of QC samples and 80% of the biological replicates were considered in the final analysis.

### 2.13 Measurement of Serum LDL‐C and Insulin

Serum insulin and LDL‐C levels were measured following the ELISA kit manufacturer's instructions (Jianglaibio, China).

### 2.14 Western Blotting

The procedures for immunoblotting hippocampal tissue proteins followed previously described methods [29]. The primary antibodies used are detailed in Table S1.

### 2.15 Statistical Analysis

Data analysis was conducted using GraphPad Prism software version 10.0 (GraphPad Software, USA) and presented as mean ± standard error of the mean (SEM). Prior to statistical analysis, data distribution was assessed for normality using the Shapiro–Wilk test. Data that did not exhibit a normal distribution were analyzed using the nonparametric Kruskal–Wallis *H* test. For data that followed a normal distribution, ANOVA was applied. A two‐way ANOVA was employed to assess the impact of HFD and S9P treatment. Tukey's or Dunn–Bonferroni post hoc tests were used to determine statistical differences among groups. Statistical significance was set at a *p* value < 0.05.

## 3 Results

### 3.1 S9P Improved Glucose Homeostasis in HFD‐Induced DACI Mice

To determine the antidiabetic effects of S9P, mice were fed a high‐fat diet with oral administration of S9P. During the experiment, the body weight in the HFD group was higher than that in ND mice. S9P treatment had no effect on the body weight at each timepoint of detection (Figure 1B, *p* > 0.05). The calorie intake of HFD + S9P mice was not significantly different from that of HFD mice at each timepoint during the 4 weeks of S9P feeding (Figure 1C, *p* > 0.05). At 120 days of HFD diet (Week 0), FBG was significantly higher in HFD group mice compared with the ND group (Figure 1D, *p* < 0.001), while S9P improved fasting glucose values after 4 weeks of supplementation (Week 4) (Figure 1D, *p* < 0.001). Similarly, S9P intervention improved glucose tolerance (Figure 1E,F, *p* < 0.001) and insulin sensitivity (Figure 1G,H, *p* < 0.001) compared with the HFD group.

### 3.2 S9P Supplementation Ameliorated Cognitive Decline in HFD‐Induced DACI Mice

**FIGURE 2:**
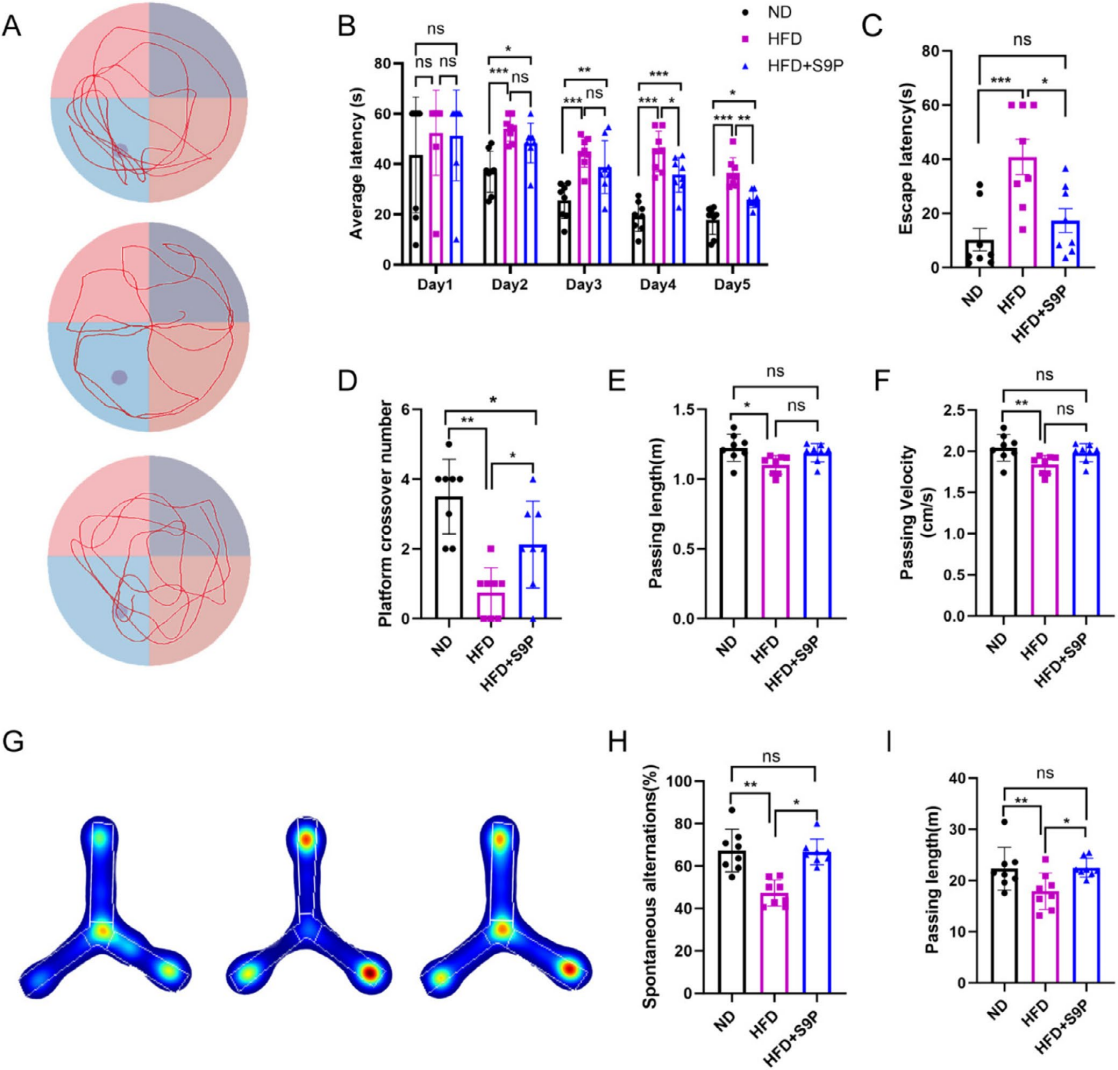
S9P administration for a month improved cognitive decline in DACI mice. (A) The representative images of the tracks of mice in the Morris Water Maze test, *n* = 8. (B) Latency to first reach the platform during training. The latency to first reach the platform area (C), the number of platform crossings (D), the total travel distance (E), and the movement speed for each group (F) on the exploration test day, *n* = 8. (G) Representative images of the tracks of mice in the Y Maze test, *n* = 8. The spontaneous alternations (H) and passing length (I) for each group. *p < 0.05, **p < 0.01, ***p < 0.001.

To assess the impact of S9P supplementation on cognitive decline induced by a high‐fat diet, we conducted water maze and Y‐maze tests, which evaluate learning, memory, and working memory. In the water maze test, the ND group exhibited a significant reduction in platform‐finding latency by the second day of training, and S9P supplementation significantly decreased the latency in the HFD group on the fourth and fifth days (Figure 2B, both *p* < 0.05). During the probe trial (sixth day), the HFD group displayed a significantly longer latency to locate the platform compared with the ND group, whereas S9P supplementation markedly reduced this latency (Figure 2A,C, *p* < 0.05). Additionally, mice in the HFD group crossed the platform fewer times compared with the ND group, while S9P supplementation significantly improved the number of platform crossings (Figure 2D, *p* < 0.05). Meanwhile, S9P supplementation had no significant effect on the swimming distance or speed in HFD mice (Figure 2E,F, *p* > 0.05). In the Y‐maze test, S9P supplementation significantly increased the spontaneous alternation percentage in the HFD group (Figure 2G,H, *p* < 0.05). The movement distance in HFD mice was significantly reduced compared with the ND group (*p* < 0.05), whereas it notably increased after S9P supplementation (Figure 2I, *p* < 0.05).

### 3.3 S9P Alleviated Neuronal Damage and Dendritic Degeneration in HFD‐Induced DACI

**FIGURE 3:**
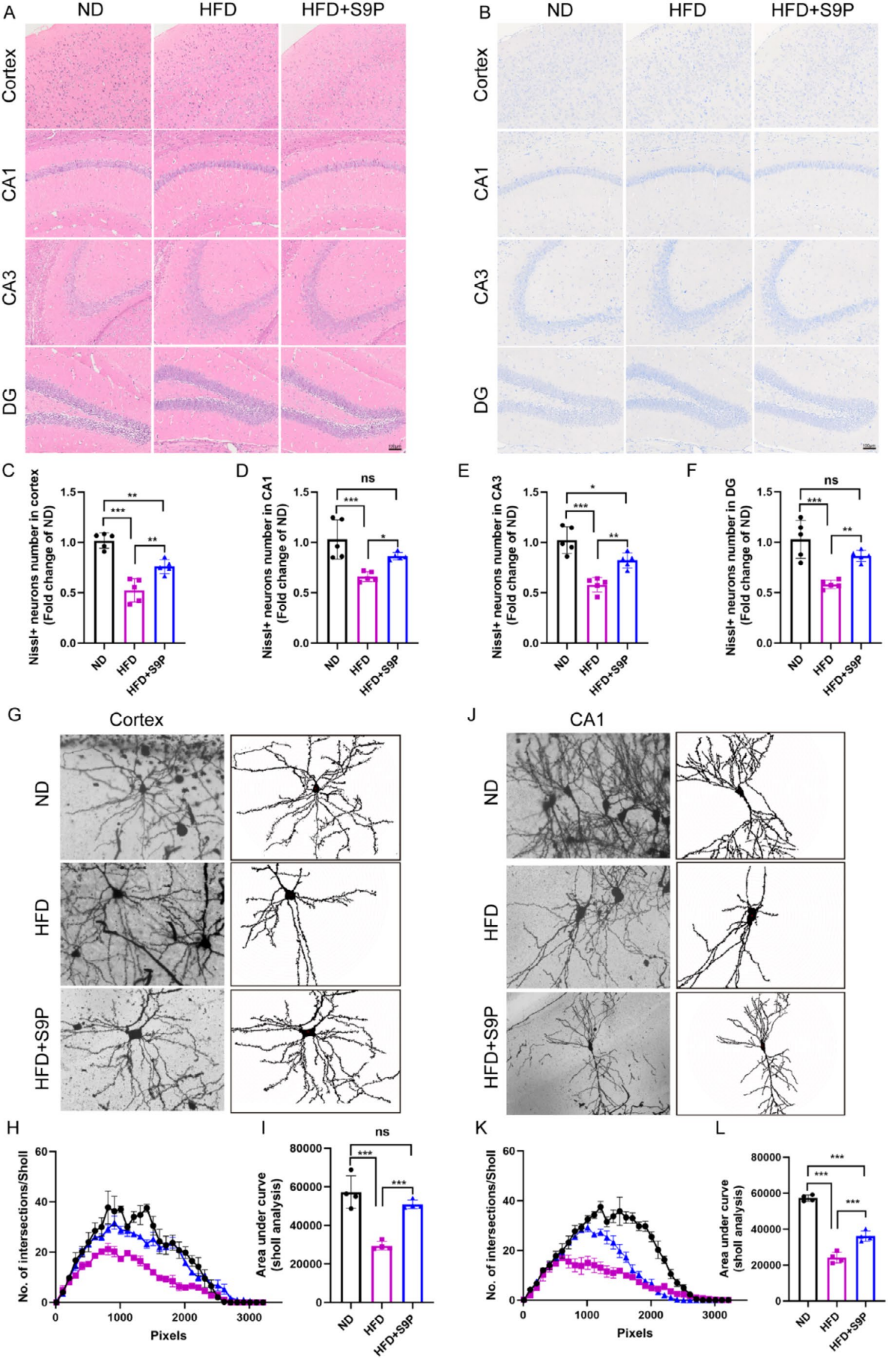
S9P administration for 1 month attenuated HFD‐induced neuronal damage and dendritic degeneration. (A) Representative image of HE staining, scale bars, 100 μm. (B) Representative image of Nissl staining (×200), scale bars, 100 μm. Nissl+ neurons in cortex (C), CA1 (D), CA3 (E), DG (F) section, *n* = 5. (G, J) Representative Golgi‐stained images of the cortex and hippocampus CA1 region along with a graphic description of Sholl analysis parameters. Statistical analysis of data obtained by Sholl analysis of Golgi‐stained neurons (H, I, K, L), *n* = 4. Data were displayed as the mean ± SEM. *p < 0.05, **p < 0.01, ***p < 0.001.

In the hippocampal regions of the high‐fat diet (HFD) group, H&E staining revealed marked disorganization and cell loss, indicating that the HFD may have caused neuronal damage or degeneration (Figure 3A). In contrast, S9P supplementation significantly mitigated these detrimental effects, which displayed a more intact and organized tissue structure than the HFD group (Figure 3A). Nissl staining further confirmed that S9P restored the reduction of neuronal density and disorganized neurons induced by HFD in the cerebral cortex and hippocampal regions (CA1, CA3, DG), demonstrating its potential neuroprotective effect (Figure 3B–F, all *p* < 0.05). Golgi staining revealed that neurons in the cortex and hippocampal CA1 region of HFD‐fed mice had significantly reduced dendritic complexity and spine density compared with those of ND mice (Figure 3G,J). Sholl analysis, used to quantify dendritic intersections at varying distances from the soma, showed that dendritic complexity was markedly diminished in HFD mice (Figure 3H,K). However, S9P supplementation significantly increased the number of intersections, suggesting that S9P exerts a protective effect on dendritic morphology (Figure 3G,H,K). Additionally, based on the Sholl analysis, the area under the curve (AUC), which quantifies overall dendritic complexity, was significantly reduced in the HFD group, indicating a simplified dendritic structure (Figure 3I,L, both *p* < 0.001). S9P supplementation significantly restored dendritic complexity in both the cortex and CA1 regions, highlighting its restorative effect (Figure 3I,L, both *p* < 0.001).

### 3.4 S9P Restored Colonic Barrier Integrity and Ameliorated Microbiome Imbalances, Insulin Resistance, and Lipid Imbalance in HFD‐Induced DACI Mice

**FIGURE 4:**
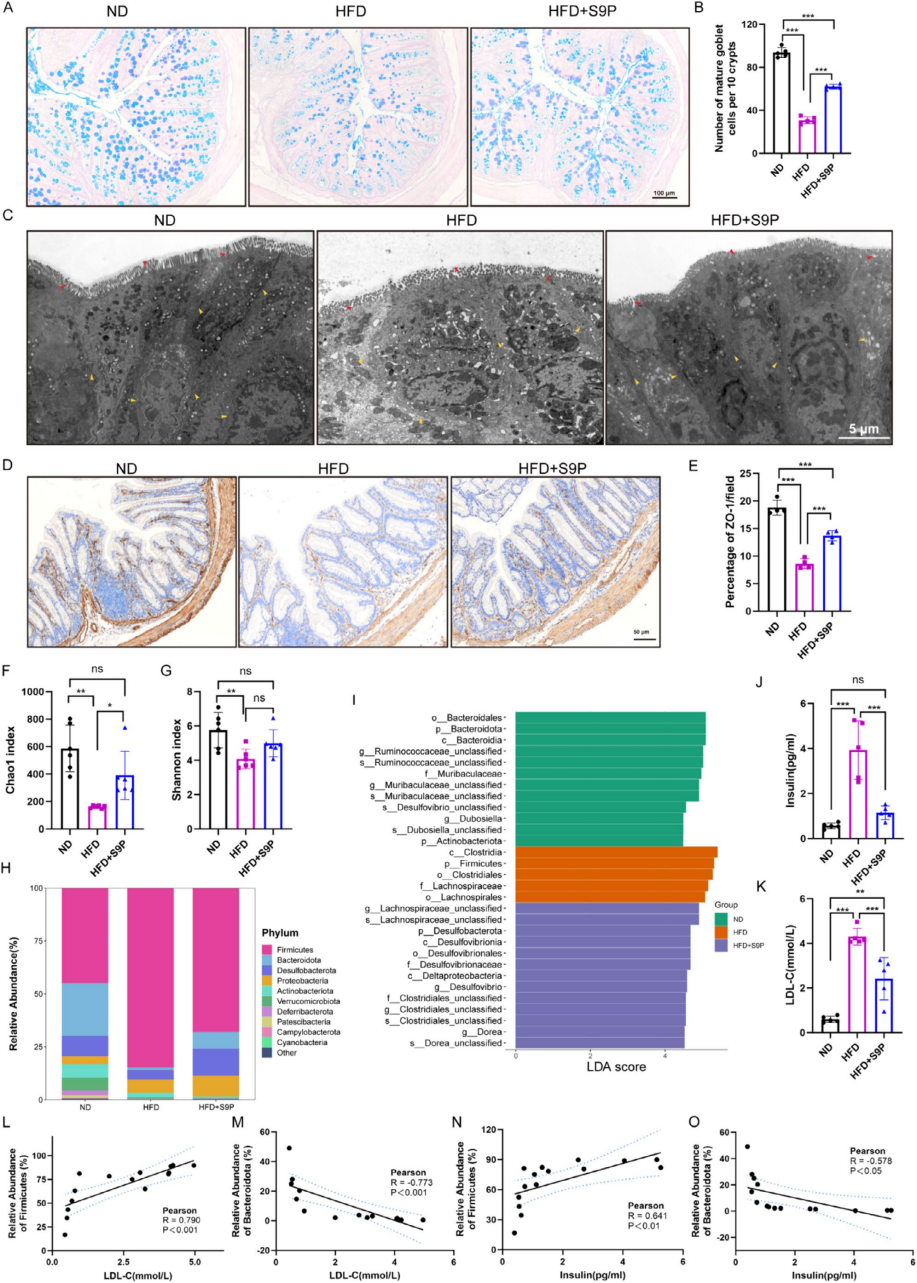
S9P was administered for one month restored colonic barrier integrity and ameliorates microbiome imbalances, insulin resistance, and lipid imbalance in HFD‐Induced DACI Mice (A) Alcian blue–periodic acid Schiff staining of colon in different groups, scale bars, 100 μm. (B) Quantification of mature goblet cells, *n* = 5. (C) Representative transmission electron microscopy images of the colon of mice in each group, intestinal villi (red arrows), tight junctions between cells (yellow arrows), scale bar: 5 μm. (D)Immunohistochemical images of colon sections stained with ZO‐1, scale bar: 50 μm. (E) Quantification of ZO‐1+ cells, *n* = 4. (F) Chao1 index, *n* = 6. (G) Shannon index, *n* = 6. (H) The abundance of dominant bacterial phyla. (I) Linear discriminant analysis (LDA) effect size (LEfSe) showed the most significantly abundant taxa‐enriched microbiome in different groups. (J, K) Serum insulin and LDL‐C levels, *n* = 5. (L, M) Correlation analysis between LDL‐C and the expression abundance of Firmicutes and Bacteroidota. (N, O) Correlation analysis between insulin and the expression abundance of Firmicutes and Bacteroidota. Values were mean ± SEM. *p < 0.05, **p < 0.01, ***p < 0.001. Abbreviations: P, phylum; c, class; o, order; f, family; g, genus.

To assess the impact of S9P on the colonic barrier in mice subjected to HFD, we found that HFD markedly reduced the number of mature goblet cells in colonic crypts and that S9P supplementation significantly restored these cell numbers (Figure 4A,B, *p* < 0.001). Under TEM, we further revealed that HFD compromised the integrity of colonic tight junctions and led to disorganized, sparse villi. In contrast, S9P supplementation preserved tight junction integrity and promoted a dense, orderly villus arrangement (Figure 4C). Additionally, S9P supplementation significantly upregulated ZO‐1 protein expression in the colon of HFD‐fed mice (Figure 4D,E, *p* < 0.001). Moreover, we found that S9P partially reversed the reduction of barrier protein occludin fluorescence intensity in the cortex of HFD mice (Figure S2A,B). Collectively, these findings suggest that S9P supplementation mitigates HFD‐induced disruption of colonic tight junctions and mucosal barrier function.

We also detected the α diversity by the Shannon and Chao1 index in order to evaluate the diversity of the gut microbiota. Compared with the ND group, the α diversity was significantly reduced in HFD mice (Figure 4F,G, both *p* < 0.05). S9P supplementation notably increased the Chao1 index (Figure 4F, *p* < 0.05). Additionally, we found that HFD induced a marked increase in Firmicutes and a reduction in Bacteroidota abundance, which were both partially reversed by S9P supplementation (Figure 4H,I). In particular, S9P reduced the abundance of Clostridium at the class level, Clostridiales and Lachnospirales at the order level, and Clostridiaceae, Lachnospiraceae, and Ruminococcaceae at the family level in Firmicutes, as well as Lachnospiraceae_NK4A136_group, Lachnoclostridium, Desulfovibrio, Clostridium, and Dorea at the genus level (Figure 4I, Figure S3A–D). S9P also upregulated bacteroidia at the class level, Bacteroidales at the order level, and Muribaculaceae at the family level in Bacteroidetes (Figure 4I, Figure S3A–D).

Additionally, we measured serum insulin and LDL‐C levels to investigate the link between microbiota and lipid metabolism (Figure 4J,K). The HFD group showed significantly elevated insulin and LDL‐C levels (both *p* < 0.001, vs. *ND group*), indicating insulin resistance and lipid imbalance in HFD mice. S9P intervention remarkably reduced these levels in DACI mice (both *p* < 0.001). Moreover, the correlation analysis revealed a positive correlation between LDL‐C/insulin and Firmicutes abundance (Figure 4L, Pearson's *R* = 0.790, *p* < 0.001; Figure 4N, Pearson's *R* = 0.641, *p* < 0.01), and a negative correlation with Bacteroidetes (Figure 4M, Pearson's *R* = −0.773, *p* < 0.001; Figure 4O, Pearson's *R* = −0.578, *p* < 0.05), suggesting that S9P improves lipid metabolism and gut microbiota balance in DACI mice.

### 3.5 S9P Modulated Metabolite Profiles and PI3K/AKT/mTOR Signaling Pathways in Mice With DACI

**FIGURE 5:**
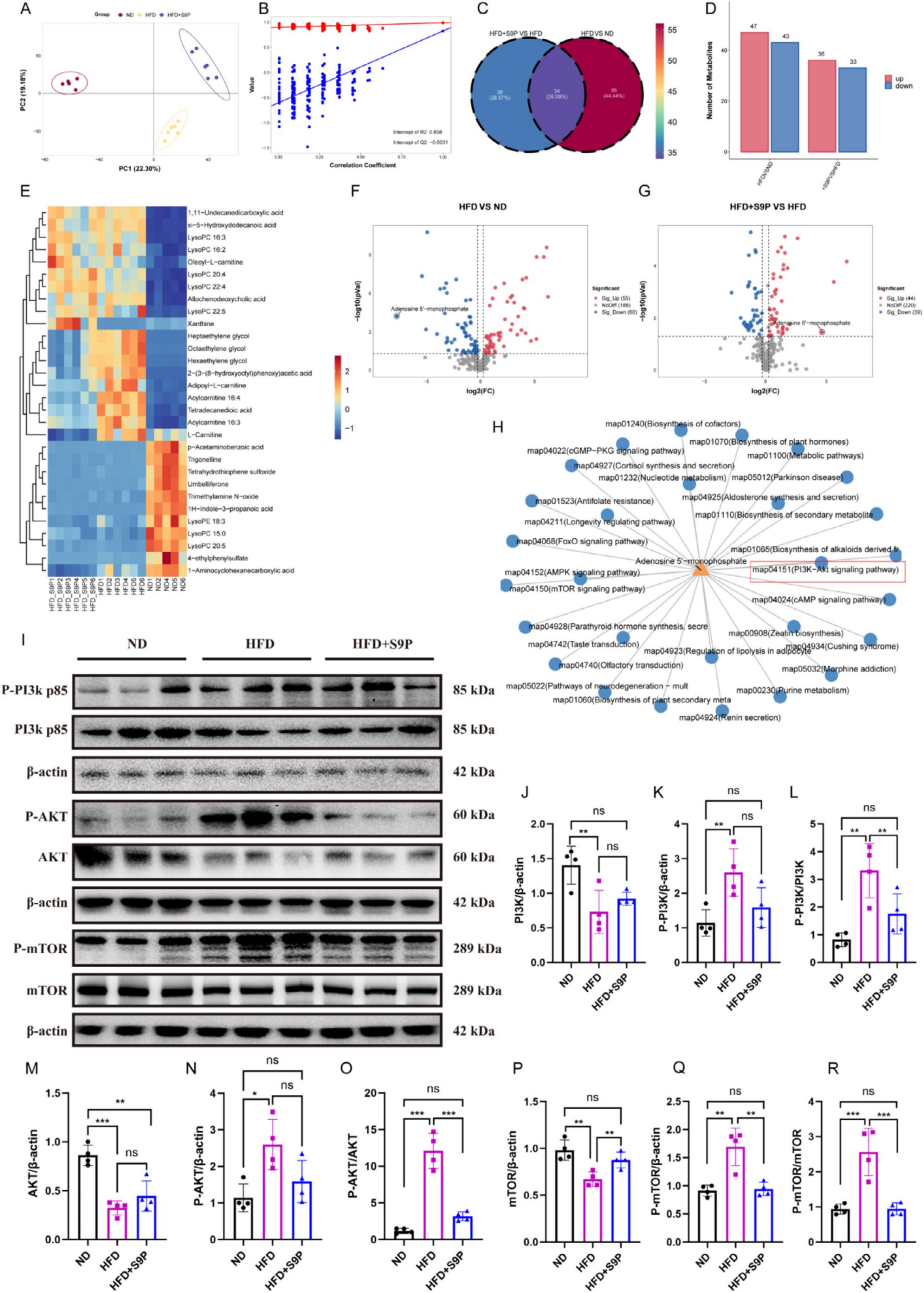
S9P Supplementation for one month modulated alterations in serum metabolites and the PI3K/AKT/mTOR signaling pathway in DACI mice. (A) Principal component analysis (PCA) of β‐diversity. (B) The permutation validation plot. (C) Venn based on the serum metabolites between HFD + S9P vs. HFD and HFD vs. ND. (D) Bar graph showing the number of upregulated (red) and downregulated (blue) metabolites in different groups. (E) Heatmap of relative abundance levels of various metabolites in different groups. Volcano plots comparing metabolite expression changes between groups, (F) HFD versus ND and (G) HFD + S9P versus HFD. (H) Pathway enrichment network centered on 5′‐adenosine monophosphate, highlighting the PI3K‐AKT signaling pathway with a red box. (I) Western blot showing the expression of P‐PI3K, PI3K, P‐AKT, AKT, P‐mTOR, mTOR. (J‐R) Quantification of P‐PI3K, PI3K, P‐AKT, AKT, P‐mTOR, mTOR expressions was normalized to β‐Actin, and the ratio of phosphorylated to nonphosphorylated forms, *n* = 4. Data are displayed as mean ± SEM. *p < 0.05, **p < 0.01 and ***p < 0.001.

To investigate the effects of S9P on the metabolome of HFD‐induced DACI mice, serum metabolomics analysis was performed. Results showed distinct metabolite clustering among the three groups, confirmed by PCA and PLS‐DA (Figure 5A,B). Differential metabolite analysis identified 90 metabolites between the HFD and ND groups, and 70 differential metabolites between the HFD + S9P and HFD groups (Figure 5C,D). The Venn diagram shows 34 common differential metabolites across these comparisons (Figure 5C). The heatmap illustrates the expression patterns of differential metabolites across the groups (Figure 5E), while volcano plots display upregulated and downregulated metabolites (*p* < 0.05, |log_2_FC| > 1). Notably, adenosine 5'‐monophosphate (AMP) was downregulated in the HFD group but upregulated after S9P intervention (Figure 5F,G), correlating with Firmicutes and Bacteroidota abundances (Figure S4A,B). Pathway enrichment analysis of AMP indicated a significant association with the PI3K‐AKT–mTOR signaling pathway following HFD and S9P intervention (Figure 5H). Figure 5I presented the expression levels of PI3K, AKT, mTOR, and their phosphorylated forms in the three groups. Compared with the ND group, the HFD group showed significantly reduced levels of PI3K, AKT, and mTOR protein in the hippocampus (Figure 5I,J,M,P), which were partially restored in the HFD + S9P group. In contrast, phosphorylated PI3K, AKT, and mTOR levels were upregulated in the HFD group but significantly downregulated after S9P intervention (Figure 5I,K,L,N,O,Q,R, all *p* < 0.05).

### 3.6 S9P Mitigated Neuronal Synaptic Damage, Astrocyte Activation, and Neuroinflammatory Responses in the Hippocampus of High‐Fat Diet‐Induced DACI Mice

**FIGURE 6:**
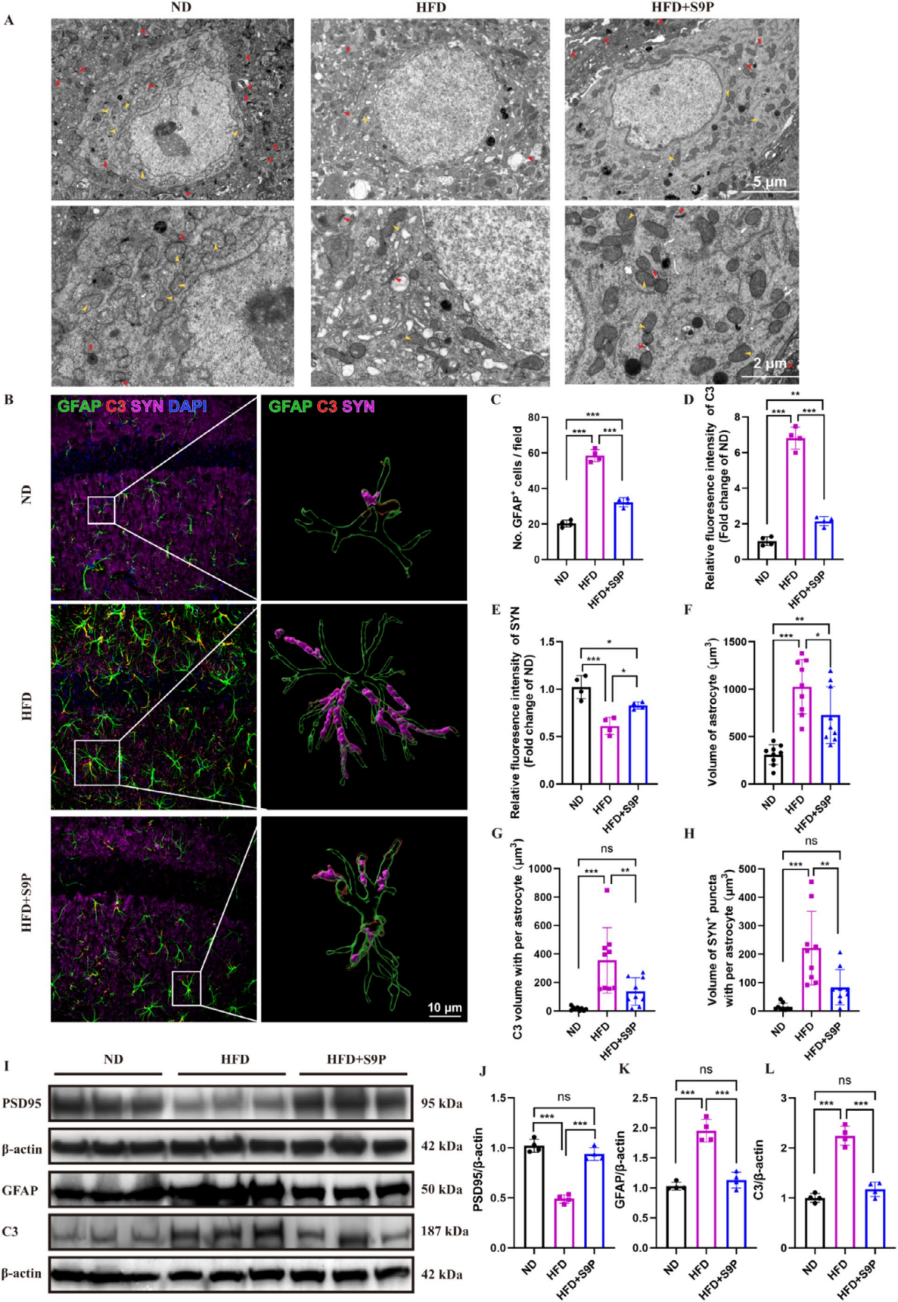
One‐month S9P intervention alleviated synaptic damage, astrocyte activation, and neuroinflammatory responses in the hippocampus of DACI mice. (A) Transmission electron microscopy images of hippocampal neurons in each group, with red arrows indicating synapses and yellow arrows indicating mitochondria. (B) Representative fluorescence images of astrocyte (green), C3(red), and SYN+ puncta (magenta) and 3D rendering of confocal stacks of astrocyte, C3, and SYN+ puncta inside astrocyte in the CA1 of different group mice. Scale bar: 10 μm. (C) Number of GFAP+ cells in the field, *n* = 4. (D, E) The quantification of C3 and SYN, *n* = 4. (F) The volume of astrocytes, (G) the C3 volume per astrocyte, (H) the SYN+ puncta volume per astrocyte, *n* = 9. (I) Western blot showing the expression of PSD95, GFAP and C3. (J‐L) Quantification of PSD95, GFAP and C3 expressions was normalized to β‐Actin, *n* = 4. Data are displayed as mean ± SEM. *p < 0.05, **p < 0.01 and ***p < 0.001.

Under TEM, we observed that hippocampal neurons in the HFD group displayed mitochondrial deformation, a lighter matrix, shortened or absent cristae (yellow arrows), reduced synapse numbers, and irregular synaptic morphology (red arrows) compared with the ND group. S9P supplementation improved mitochondrial integrity and increased synapse numbers (Figure 6A). As shown in Figure 6B, the HFD group exhibited a significant increase in GFAP‐positive cell count and C3 expression in the hippocampus, along with a marked downregulation of SYN expression (Figure 6C–E, all *p* < 0.05). The 3D reconstruction showed that astrocytes in the HFD group were activated, with increased volume, elevated C3 expression, and SYN engulfment (Figure 6F–H, all *p* < 0.05). S9P treatment reduced GFAP‐positive cells, C3 expression, and increased SYN expression (Figure 6C,D, both *p* < 0.001). 3D reconstruction further indicated reduced astrocyte volume, C3, and SYN engulfed by astrocytes after S9P intervention (Figure 6F–H, all *p* < 0.05). Western blot analysis confirmed that S9P restored the hippocampus's PSD95, GFAP, and C3 levels (Figure 6I–L, all *p* < 0.001 vs. *HFD group*). These results indicate that a high‐fat diet induces astrocyte activation, synaptic structural damage, and elevated inflammatory markers, while S9P intervention effectively reverses these changes, suggesting a protective role of S9P against neuroinflammation and synaptic damage in the DACI model.

## 4 Discussion

The gut‐brain axis presents promising therapeutic targets for HFD‐induced DACI. In this study, S9P supplementation significantly alleviated cognitive deficits, improved lipid metabolism, and reduced neuronal damage in DACI mice. It also preserved the integrity of the colonic mucosal barrier. Additionally, S9P was able to modulate the gut microbiota, specifically causing a decrease in Firmicutes abundance and an increase in Bacteroidetes, thus contributing to a more balanced microbiome. Moreover, S9P was found to promote metabolic homeostasis, activate the PI3K/AKT/mTOR pathway, reduce hippocampal astrocyte activation, and synaptic injury in HFD mice. Collectively, these results suggest that S9P exerts protective effects against HFD‐induced cognitive impairment, possibly through modulation of the gut–brain axis and the PI3K/AKT/mTOR pathway. Our findings indicate that S9P could be considered an effective therapeutic candidate for DACI.

Studies have demonstrated that PAHSAs can downregulate blood glucose levels, improve glucose tolerance, and enhance insulin sensitivity in HFD mice [30, 31]. Among endogenous PAHSAs, 9‐PAHSA is the most abundant and plays a crucial role in regulating lipid metabolism [32]. Of its stereoisomers, S9P demonstrates a higher affinity for the GPR40 receptor than R‐9‐PAHSA. This property endows S9P with superior biological activity in promoting glucose‐stimulated insulin secretion by pancreatic beta cells and enhancing glucose uptake [28]. In consistency with these researches, our results confirmed that S9P effectively improves fasting blood glucose, glucose tolerance, and insulin resistance in DACI model mice.

The intestinal microbiota and barrier are crucial components of the gut–brain axiso [33, 34]. It has been reported that dysbiosis of the microbiota can disrupt metabolic processes, compromise blood–brain barrier integrity, and impair neuronal survival and synaptic plasticity [33]. Conversely, a balanced microbiota supports epithelial cell integrity and promotes mucus layer formation, helping to block harmful substances from crossing the barrier, thereby improving inflammation or metabolic imbalance in the body [35]. A recent study indicated that PAHSAs' metabolic benefits are closely associated with the gut microbiota in HFD‐fed mice [25]. In this study, we observed that S9P enhanced gut microbial diversity in HFD‐fed mice and balanced the Firmicutes/Bacteroidetes ratio. Another study also demonstrated that increasing Bacteroidetes abundance could improve intestinal barrier function and alleviate HFD‐induced cognitive impairment in mice [36]. Interestingly, our findings revealed a positive correlation between LDL‐C, insulin, and Firmicutes abundance, with an inverse relationship noted for Bacteroidetes. Turnbaugh et al.'s study demonstrated that Firmicutes could modulate intestinal hormone secretion and influence insulin sensitivity in an obese mouse model [37]. Additionally, alterations in the gut microbiome impact intestinal barrier function and affect lipid metabolism and absorption, contributing to dyslipidemia [37]. The above results suggested that intestinal microorganisms can modulate serum metabolic profiles by influencing insulin sensitivity, lipid absorption, and intestinal barrier function.

In T2DM, serum metabolic changes primarily manifest as disruptions in carbohydrate, lipid, and amino acid metabolism, with elevated levels of several key metabolites, including serum insulin, free fatty acids (FFAs), LDL‐C, and branched‐chain amino acids (BCAAs) [38, 39]. These metabolic abnormalities are closely linked to dysregulated intracellular energy metabolism, particularly reduced AMP levels and downregulation of AMP‐activated protein kinase (AMPK) activity [40]. AMP, a nucleotide derivative of adenosine, is a crucial metabolic intermediate and energy regulatory molecule in cells [41]. In T2DM, impaired cellular energy metabolism decreases AMP levels, leading to a reduced AMP/ATP ratio, which inhibits AMPK activity and consequently impairs fatty acid oxidation and glucose uptake, thus aggravating metabolic syndrome symptoms [42]. In cellular metabolism regulation, the PI3K/AKT pathway and the AMPK pathway often exhibit an antagonistic relationship [43, 44]. Under high‐energy conditions, the PI3K/AKT/mTOR pathway is more active, while AMPK remains largely inactive conditions [45]. Our results indicated that in DACI mice, AMP correlated with intestinal microbiota alternation after S9P intervention. Meanwhile, S9P decreased PI3K/AKT/mTOR pathway in the hippocampus. Therefore, our findings indicated that S9P protects cognitive function possibly by modulating AMP levels and PI3K/AKT/mTOR pathway, which is linked to the microbiota.

Diabetes induced abnormalities in glucose and lipid metabolism may trigger astrocyte activation. Granatiero et al.'s study showed that elevated p‐mTOR levels can promote astrocyte activation [46]. Astrocyte activation and upregulation of C3 are key factors contributing to neurodegenerative diseases and neurological impairment [47]. Upon activation, astrocytes undergo notable morphological changes, such as thicker and elongated processes with increased branching and cell volume [48, 49]. These structural changes are usually accompanied by enhanced phagocytic function, thus enabling astrocytes to effectively clear cell debris, pathogens, and other damaging agents [50]. However, excessive phagocytosis may inadvertently target healthy synapses, thereby impairing neuronal function [51]. This phenomenon is particularly evident in Alzheimer's disease, where overactive astrocytes mistakenly engulf healthy synapses alongside pathological materials [51]. In addition, activated astrocytes markedly upregulate complement component C3 expression [47]. Elevated C3 levels activate the complement system, recruiting immune cells to damaged areas via molecules like C3a and C5a, which intensify the inflammatory response [52]. C3 binding to receptors (such as C3aR and C5aR) further activates microglia and other immune cells, prompting them to release additional proinflammatory factors, further aggravating the neuroinflammatory environment [52]. In this study, we found that S9P played a protective role in neuronal synapses possibly by reducing the activation level of astrocytes and C3 expression.

In summary, the current study reported that S9P mitigates neuroinflammation and synaptic dysfunction in DACI by modulating gut dysbiosis. Overall, these findings provide the first evidence that S9P can improve HFD‐induced cognitive deficits through the gut–brain axis, offering new insights into the treatment of DACI.

## Ethics Statement

Animal experiments were conducted with the approval of the Department of Laboratory Animal Science, Fudan University (Approval Number: 202111012S). All procedures were carried out in strict accordance with the Guide for the Care and Use of Laboratory Animals issued by the Ministry of Science and Technology of China.

## Conflicts of Interest

The authors declare no conflicts of interest.

## Supporting information


Appendix S1

## Data Availability

The data that support the findings of this study are available from the corresponding author upon reasonable request.
