# Supplementary material for: S‐9‐PAHSA Protects Against High‐Fat Diet‐Induced Diabetes‐Associated Cognitive Impairment via Gut Microbiota Regulation
Source: CNS Neurosci Ther. 2025 May 5;31(5):e70417. doi: 10.1111/cns.70417 (PMC12052735; doi:10.1111/cns.70417)
Supplement: Supplementary file 1 — Appendix S1 [file CNS-31-e70417-s001.docx]

**Supplementary Materials**

**Table S1. Primary antibodies for western blotting, IHC, and IF analysis**

| Antibody name | Source | Catalog number |
| --- | --- | --- |
| PI3K | Cell Signaling Technology | 4257T |
| P-PI3K | Cell Signaling Technology | 4288T |
| AKT | Proteintech | 10176-2-AP |
| P-AKT | Cell Signaling Technology | 4058S |
| mTOR | Cell Signaling Technology | 2983T |
| P- mTOR | Cell Signaling Technology | 5536T |
| ZO-1 | Abcam | ab216880 |
| Occludin | Abcam | ab216327 |
| GFAP | Cell Signaling Technology | 3670S |
| C3 | Invitrogen | PA1-29715 |
| SYN | Abcam | ab32127 |
| PSD95 | Cell Signaling Technology | 3450T |
| β-actin | Cell Signaling Technology | #3700 |

**Supplementary figures**

**
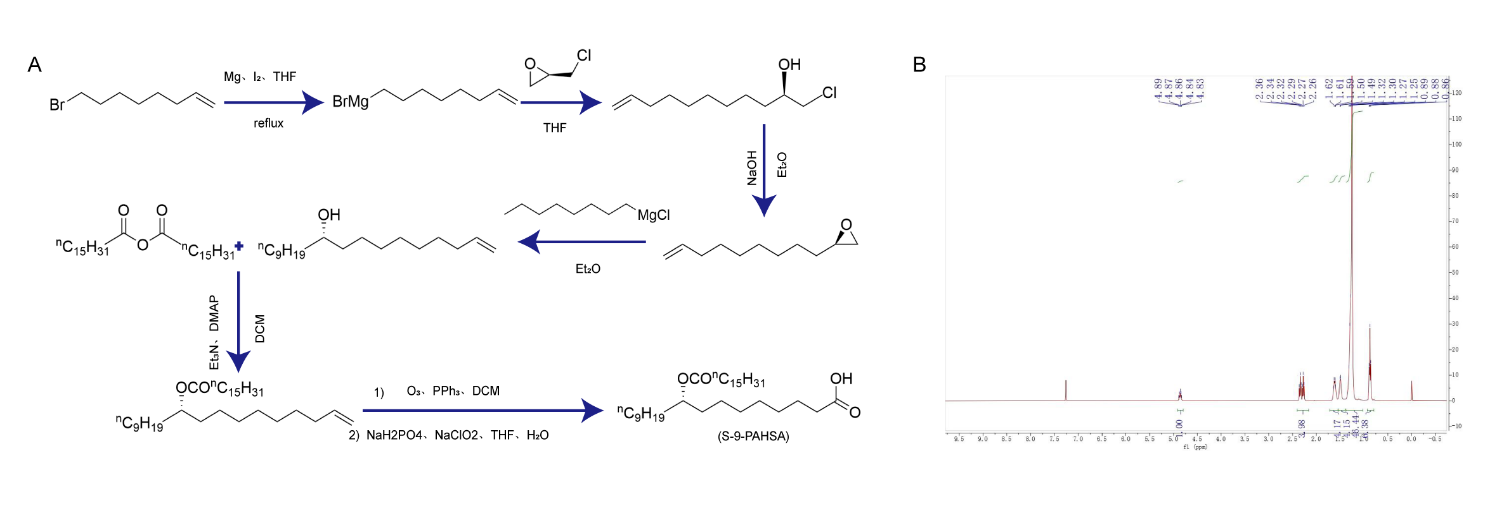
**

**Figure S1.** Preparation method and synthesis process and identification of S9P.

**
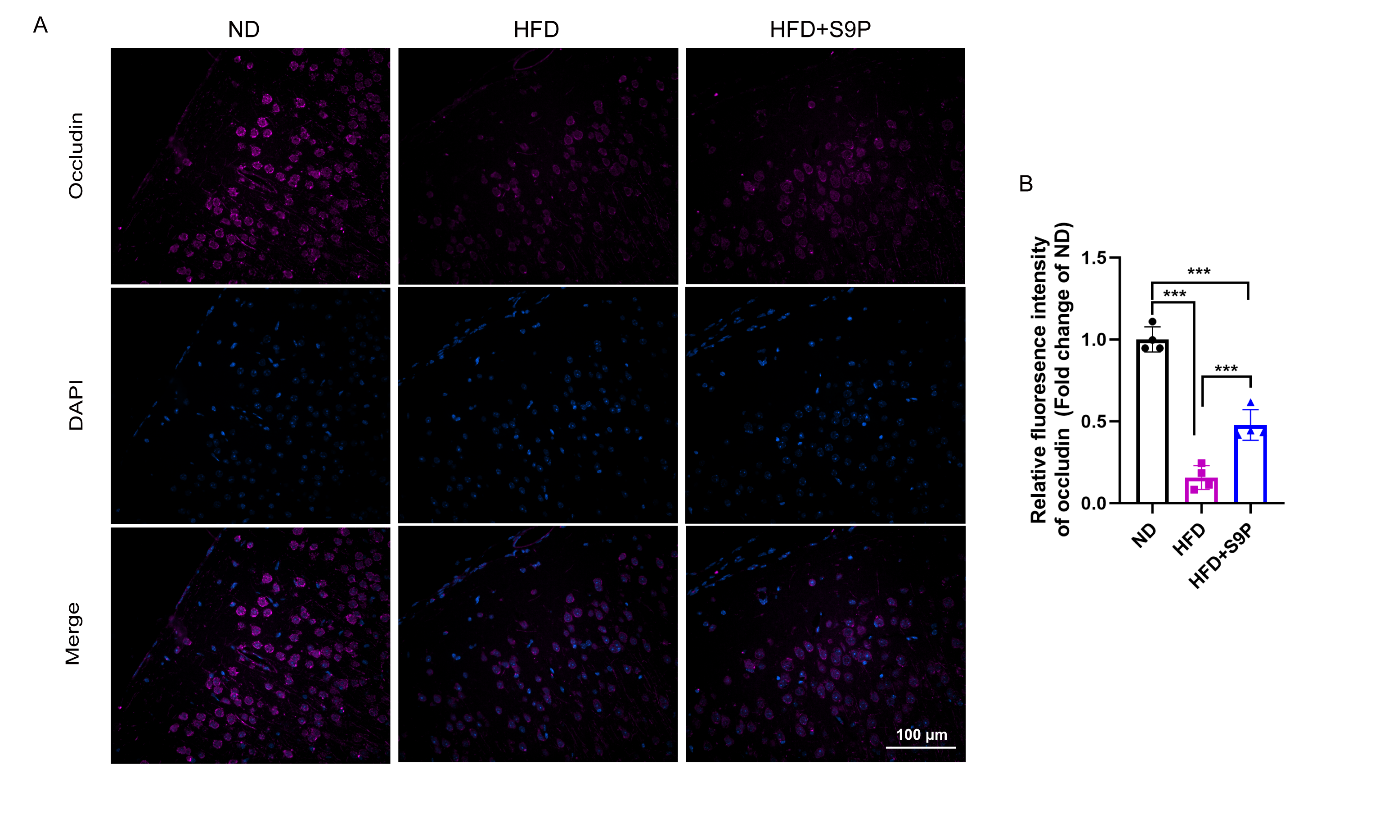
**

**Figure S2.** S9P rescued the loss of occludin in the cortex of DACI mice. (A) Representative fluorescence images of occludin in cortex. (B) quantification of C3 and SYN, n=4. Data was displayed as the mean ± SEM. ****P*<0.001.

**
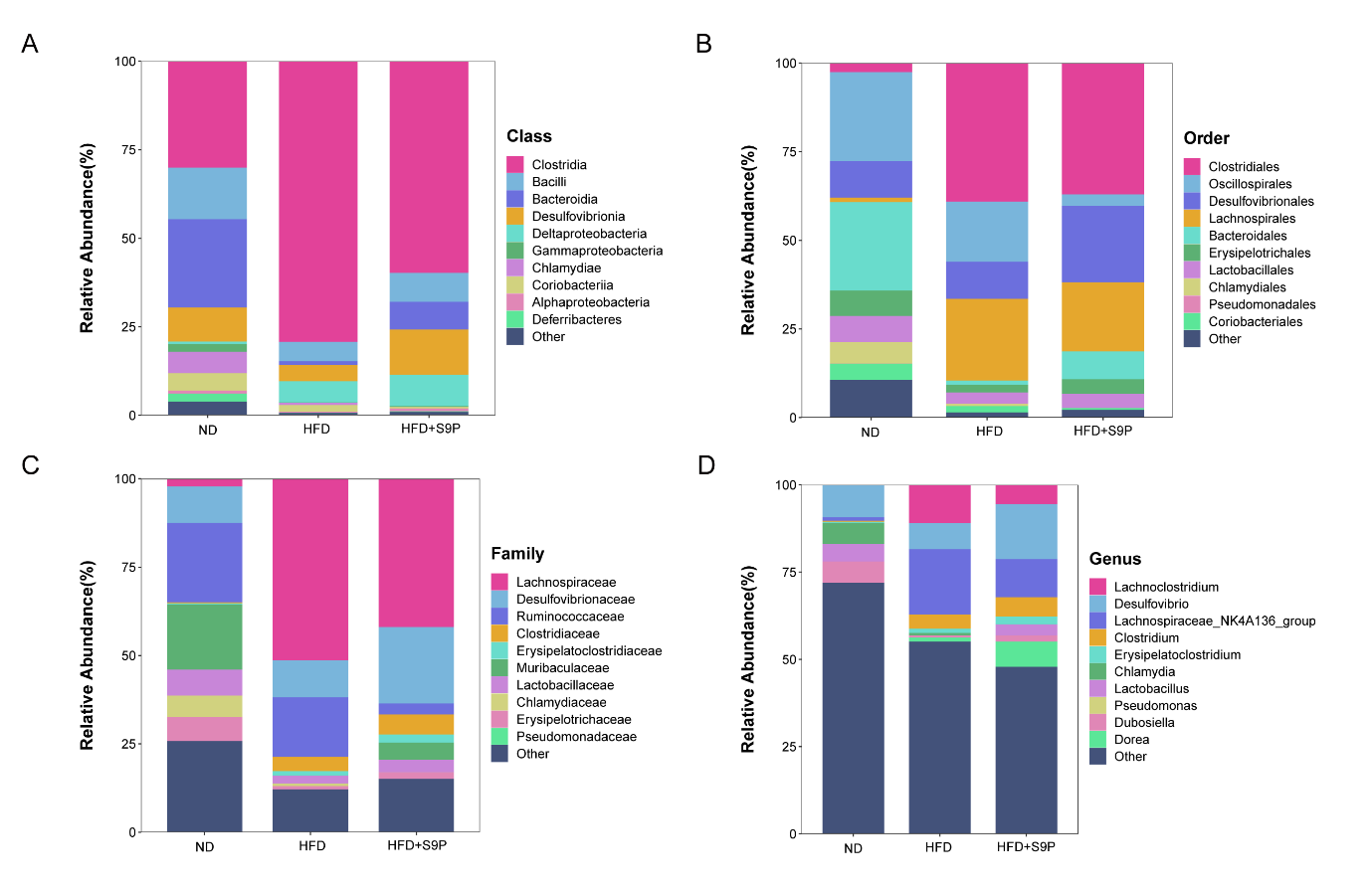
**

**Figure S3.** S9P modulated gut microbiota by reducing firmicutes and enhancing bacteroidota abundance. (A) The abundance of dominant bacterial class. (B) The abundance of dominant bacterial order. (C) The abundance of dominant bacterial family. (D) The abundance of dominant bacterial genus.


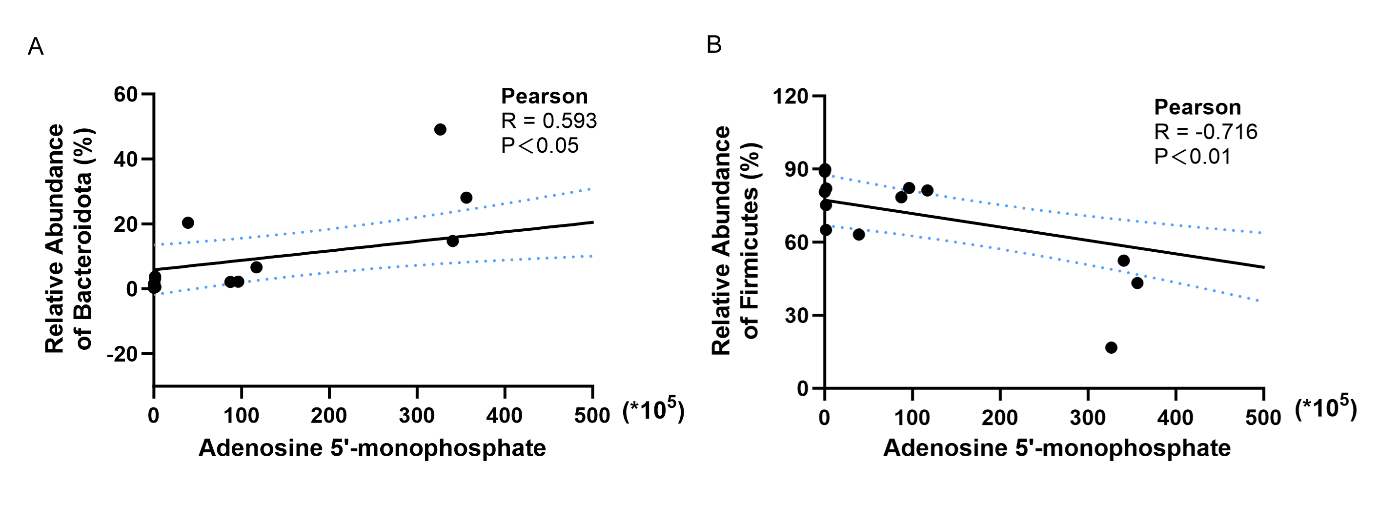


**Figure S4.** Correlation Between AMP Levels and Relative Abundance of Bacteroidota and Firmicutes in Gut Microbiota. (A) Correlation analysis between AMP and the expression abundance of Bacteroidota. (B) Correlation analysis between AMP and the expression abundance of Firmicutes.
